# Immune Signatures in Patients with Psoriasis Vulgaris of Blood-Heat Syndrome: A Systematic Review and Meta-Analysis

**DOI:** 10.1155/2016/9503652

**Published:** 2016-05-04

**Authors:** Xin Li, Qing-qing Xiao, Fu-lun Li, Rong Xu, Bin Fan, Min-feng Wu, Min Zhou, Su Li, Jie Chen, Shi-guang Peng, Bin Li

**Affiliations:** ^1^Department of Dermatology, Yueyang Hospital of Integrated Traditional Chinese and Western Medicine, Shanghai University of Traditional Chinese Medicine, Shanghai 200437, China; ^2^Institute of Dermatology, Shanghai Academy of Traditional Chinese Medicine, Shanghai 201203, China; ^3^Department of Dermatology, Beijing Chao-Yang Hospital, Capital Medical University, Beijing 100020, China

## Abstract

*Objective*. To determine whether immunological serum markers IFN-*γ*, IL-4, IL-17, IL-23, IL-6, TNF-*α*, and IL-10 are elevated or decreased in patients compared with healthy controls.* Methods*. A complete search of the literature on this topic within the past 30 years was conducted across seven databases. Seventeen studies including 768 individuals were identified. Differences in serum marker levels between subjects and controls were pooled as MDs using the random-effects model.* Results*. The pooled MDs were higher in patients than in healthy controls for IFN-*γ* (MD 24.9, 95% CI 12.36–37.43), IL-17 (MD 28.92, 95% CI 17.44–40.40), IL-23 (MD 310.60, 95% CI 4.96–616.24), and TNF-*α* (MD 19.84, 95% CI 13.80–25.87). Pooled IL-4 (MD −13.5, 95% CI −17.74–−9.26) and IL-10 (MD −10.33, 95% CI −12.03–−8.63) levels were lower in patients.* Conclusion*. The pooled analyses suggest that levels of IFN-*γ*, IL-17, IL-23, and TNF-*α* are significantly elevated and that levels of IL-4 and IL-10 are significantly decreased in sera of patients with psoriasis vulgaris of blood-heat syndrome. Measuring progression of blood-heat syndrome of psoriasis vulgaris will require additional high-quality data, with a low risk of bias and adequate sample sizes, before and after antipsoriatic therapy.

## 1. Introduction

Psoriasis is a chronic immune-mediated skin disease that affects approximately 2–4% of the population in Western countries [1]. Patients with psoriasis may present with the pustular, guttate, arthritic, or erythrodermic variants and may have itching or painful lesions that negatively affect the quality of life. The understanding of the pathogenesis of psoriasis has improved, as has the understanding of the cellular components (mainly keratinocytes and T lymphocytes) involved in psoriasis, and the cytokines produced by the main Th lymphocyte subsets are now known to play a decisive role in pathogenesis [2]. Current evidence suggests that psoriasis is a T-cell-mediated disease driven at least in part by a positive feedback loop from activated T-cells to antigen-presenting cells (APCs) that is mediated by IFN-*γ*, IL-1, and tumor necrosis factor-*α* (TNF-*α*) [3, 4]. It has been shown that the Th1-Th2-Th17 balance is likely a key functional and genetic determinant of psoriasis [4].

However, current treatments remain unsatisfactory and burdensome, and they often do not meet patients' expectations [5]; thus, therapies collectively called alternative therapies are commonly used. One observational study revealed that 51% of patients with psoriasis opted to use alternative therapies [6]. Traditional Chinese medicine (TCM), one type of alternative therapy, has been used to treat human diseases for more than 2000 years in China. Over the course of history, physicians practicing TCM have accumulated a tremendous amount of knowledge and experience in treating psoriasis. TCM prescribes treatment for psoriasis vulgaris based on syndrome differentiation. According to TCM, the syndromes can be divided into three main categories: blood-heat syndrome, blood-stasis syndrome, and blood-dryness syndrome. Correspondingly, clearing heat and cooling blood, promoting blood circulation to dissipate blood stasis, and adding moisture to reduce blood dryness comprise the treatment principles for the three syndromes of psoriasis vulgaris [7, 8]. The distribution of the three syndromes has been shown to be closely correlated with disease stage: the blood-heat syndrome is the most common at the active stage; the blood-dryness syndrome is the most common at the resting and regressive stages; and the blood-stasis syndrome is the most common at the resting stage [9]. Several investigators have searched for immunological markers of blood-heat syndrome, not only in skin lesions, but also in the circulatory system, using them to measure disease severity or to quantify treatment response. However, the data on serum levels of immunological markers in patients compared with controls are contradictory; some authors have reported elevated levels, whereas others have reported conflicting results. The studies to date have used small sample sizes or have investigated different markers to assess immune status; moreover, measurement of immunological serum markers is often not their primary objective.

We performed a systematic review and meta-analysis to determine whether seven well-known immunological serum markers (IFN-*γ*, IL-4, IL-17, IL-23, IL-6, TNF-*α*, and IL-10) are elevated or decreased in patients with psoriasis vulgaris of blood-heat syndrome compared with controls.

## 2. Materials and Methods

### 2.1. Eligibility Criteria

Inclusion and exclusion criteria were determined before the search was conducted. We included human studies comparing patients with psoriasis vulgaris of blood-heat syndrome with healthy controls, in which one or more of the following immunological markers was measured in the serum: IFN-*γ*, IL-4, IL-17, IL-23, IL-6, TNF-*α*, and IL-10. If several studies reported results from the same study population, the most complete report was included. Case reports and letters were excluded.

### 2.2. Data Sources and Searches

To identify relevant psoriasis vulgaris of blood-heat syndrome studies that included immunological markers, three reviewers (X. L., Q. Q. X, and F. L. L.) systematically searched MEDLINE, Embase, Cochrane Central Register of Controlled Trials, China National Knowledge Infrastructure database (CNKI), Chinese Scientific Journals Full-Text Database (CQVIP), Wanfang Data Knowledge Service Platform, and Chinese Biomedical Literature Service System (SINOMED). Papers published in English or Chinese and dated from January 1980 to May 2015 were included in this study.

The main descriptors adopted in the search strategy for primary studies were psoriasis, blood-heat syndrome, IFN-*γ*, IL-4, IL-17, IL-23, IL-6, TNF-*α*, and IL-10.

The search strategy adopted in the MEDLINE database via PubMed, which was adapted for the other databases analyzed, is presented as follows: psoriasis[tw] AND blood-heat syndrome[tw] AND (interferon-*γ*[tw] OR IFN-gamma[tw] OR IFN-*γ*[tw] OR interleukin-4[tw] OR il-4[tw] OR interleukin-17[tw] OR il-17[tw] OR interleukin-23[tw] OR il-23[tw] OR interleukin-6[tw] OR il-6[tw] OR tumor necrosis factor^*∗*^[tw] OR tnf[tw] OR interleukin-10[tw] OR il-10[tw]) NOT (animals[mesh] NOT humans[mesh]) NOT (case reports[pt] OR letter[pt]).

### 2.3. Study Selection

**Figure 1:**
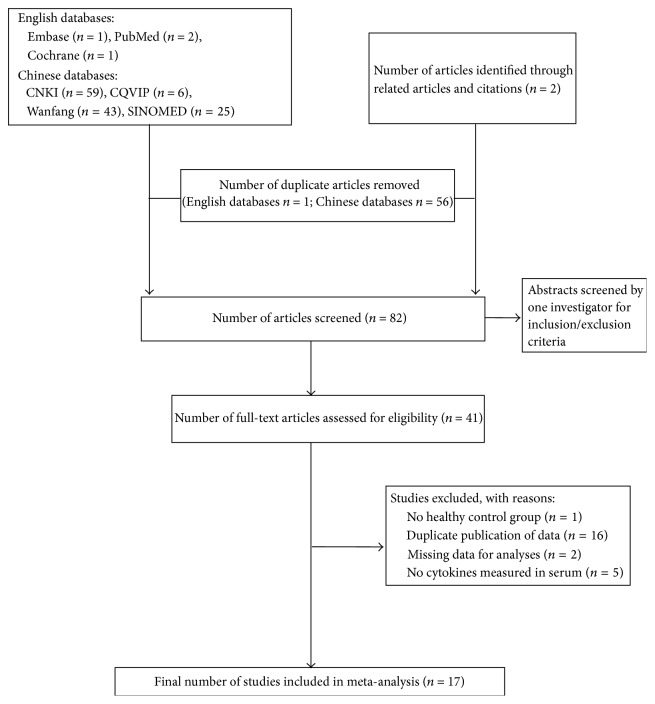
Flowchart of literature search and study selection.

To determine eligibility for inclusion in the review, we screened all titles and abstracts for the following criteria: analyses comparing immunological marker profiles of patients with psoriasis vulgaris of blood-heat syndrome with those of control groups. There were no limitations on the study design, participant's age, gender, or nationality. The selection criteria for inclusion were as follows: (i) human-only studies; (ii) original data; (iii) a healthy control group; and (iv) provision of means and confidence intervals (CIs) for immunological serum markers. We identified 138 articles in the initial search (Figure 1). Through manual review of the citations from these articles, we identified 2 additional articles. After removing 57 duplicate articles and reading 82 individual abstracts, we identified 41 original studies that were eligible for inclusion criteria assessment. After reviewing the full text of these 48 studies, we excluded 24 articles for the following reasons: no healthy control group, duplicate publication of data, missing data from analyses, and no cytokines measured in serum. In the end, we selected 17 studies that met the inclusion criteria for this systematic review [10–26]. A flowchart of the search process is presented in Figure 1.

### 2.4. Data Extraction and Quality Assessment

Three reviewers independently collected descriptive data for each included study: (i) the first author; (ii) study characteristics (i.e., year, duration, country, and setting); (iii) characteristics of participants (i.e., mean age, male-to-female ratio, numbers of case and control subjects, duration, and mean PASI of cases); and (iv) outcome characteristics (i.e., the mean of immunological serum markers of psoriasis vulgaris blood-heat syndrome along with the standard deviation (SD) and whether results were from primary analysis of the study or were adjusted for comorbidities).

The Newcastle-Ottawa Scale [27] was used to assess study quality. It categorizes studies by three dimensions including selection, comparability, and exposure for case-control studies and selection, comparability, and outcome for cohort studies. Selection included four items, comparability included one item, and exposure included three items.

### 2.5. Data Synthesis and Analysis

The primary outcome was the identification of differences in mean serum levels of immunological markers between patients with psoriasis vulgaris of blood-heat syndrome and healthy controls for each study (Table 2). The degree of heterogeneity between studies was assessed using the *I* ^2^ test. An *I* ^2^ value > 50% was considered to indicate substantial heterogeneity. In this case, DerSimonian and Laird random-effects models were considered to compute the global MD. The between-study heterogeneity was not substantial (*I* ^2^ < 50%) and the fixed-effect model was suitable. Publication bias was investigated graphically by using funnel plots and was statistically assessed via Egger's regression. The methods and findings of the present review have been reported following the Meta-Analysis of Observational Studies in Epidemiology group guidelines and checklist [28]. The Cochrane Collaboration Software Review Manager 5.2 was used for meta-analysis (http://ims.cochrane.org/revman). Egger's regression was performed using STATA version 10.0 (STATA Corp., College Station, TX, USA).

## 3. Results

### 3.1. Study Selection

Of a total of 139 titles, the full text of 41 potentially relevant studies was reviewed to confirm their eligibility. Among these 41 studies, 24 were excluded, including one with no healthy control group, 23 with duplicate publication of data, two with missing data for analyses, and five with no measurement of cytokines in serum. In total, 17 trials met the inclusion criteria (Figure 1).

### 3.2. Study Characteristics

The 17 selected studies included data on 768 individuals (443 patients with psoriasis vulgaris of blood-heat syndrome and 325 healthy controls). All of these seventeen studies were conducted in China; sixteen were published in Chinese and one was published in English. The age and male-to-female ratios of patients with psoriasis vulgaris of blood-heat syndrome and healthy controls were comparable (Table 1). In total, 59% of the studies reported a Psoriasis Area and Severity Index (PASI). Of these, 90% of the patients were from studies reporting a mean PASI > 10, indicating that the majority of the studies included patients with severe disease. The reported Newcastle-Ottawa Scale scores were between 3 and 7, as shown in Table 1. Specifically, fifteen of the studies were deemed as medium-quality (4 to 6 stars), one [20] was deemed as poor-quality (<4 stars), and another [26] was deemed as high-quality (7 or >7 stars).

### 3.3. Outcomes

**Figure 2:**
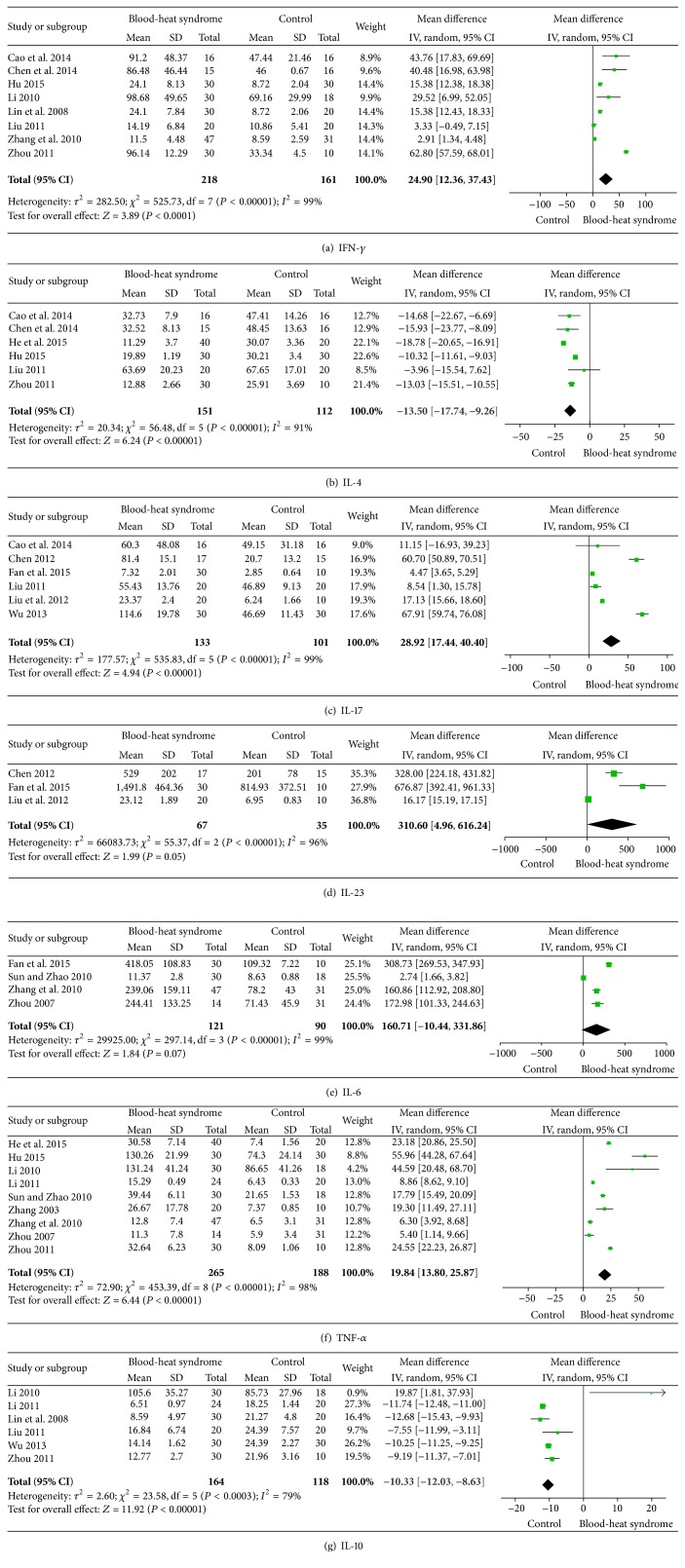
Meta-analysis of serum IFN-*γ*, IL-4, IL-17, IL-23, IL-6, TNF-*α*, and IL-10 levels in patients with psoriasis vulgaris of blood-heat syndrome. The mean difference (MD) in IFN-*γ*, IL-4, IL-17, IL-23, IL-6, TNF-*α*, and IL-10 levels of patients with psoriasis compared with controls. The point estimate (center of each green square) and statistical size (proportional area of the square) are represented. Horizontal lines indicate 95% confidence intervals. The subtotal and total MDs (diamonds) were calculated using random-effects models.

Meta-analysis of the results for all markers, including IFN-*γ*, IL-4, IL-17, IL-23, IL-6, TNF-*α*, and IL-10, for subjects with psoriasis vulgaris of blood-heat syndrome revealed significant between-study heterogeneity (*I* ^2^ > 75%) with random-effects modeling. The MD for studies analyzing IFN-*γ* was 24.9 (95% CI 12.36–37.43), indicating a significant difference in serum IFN-*γ* between the 218 patients with psoriasis vulgaris of blood-heat syndrome and 161 controls (Figure 2(a)). Six studies reported plasma IL-4 levels, in 151 patients and 112 controls.  Figure 2(b) shows that a significantly lower level of IL-4 was observed in patients with psoriasis vulgaris of blood-heat syndrome, with a pooled MD of −13.5 (95% CI −17.74–−9.26). Six studies (including 133 patients and 101 controls) showed an elevated MD for IL-17 (28.92, 95% CI 17.44–40.40) (Figure 2(c)). The mean IL-23 across studies was significantly elevated in the 67 patients compared with the 35 controls (MD 310.60; 95% CI 4.96–616.24) (Figure 2(d)). Pooling of IL-6 levels resulted in a small, positive but not statistically significant MD between 121 patients with psoriasis vulgaris of blood-heat syndrome and 90 healthy controls (160.71; 95% CI −10.44–331.86) (Figure 2(e)). In total, nine articles including 265 patients and 188 controls showed a significantly elevated MD for TNF-*α* between psoriasis and controls (MD 19.84, 95% CI 13.80–25.87) (Figure 2(f)). The combined MD for IL-10 was decreased in 164 patients compared with 118 controls (MD −10.33, 95% CI −12.03–−8.63) (Figure 2(g)).

### 3.4. Assessment of Publication Bias

The funnel plots for IFN-*γ*, IL-4, IL-17, IL-23, IL-6, TNF-*α*, and IL-10 showed evidence of asymmetry (Figure S1 in Supplementary Material available online at http://dx.doi.org/10.1155/2016/9503652; see MOOSE checklist).

Egger's test confirmed the presence of publication bias for IFN-*γ* (9.29, 95% CI 2.30–16.28), IL-4 (−17.74, 95% CI −28.06–−7.42), IL-17 (10.90, 95% CI 2.53–19.28), IL-23 (8.54, 95% CI 1.68–15.42), TNF-*α* (9.78, 95% CI 5.74–13.83), and IL-10 (−14.61, 95% CI −23.40–−5.81). There appeared to be no publication bias for IL-6 (7.06, 95% CI −1.88–16.01).

## 4. Discussion

### 4.1. Summary of Evidence

**Figure 3:**
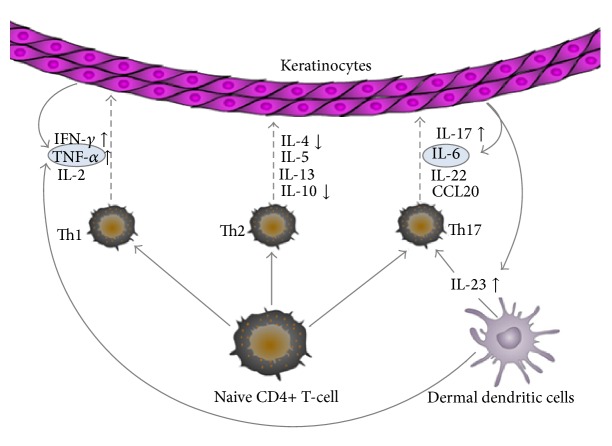
A simplified model depicting the role of the immunological markers with psoriasis vulgaris of blood-heat syndrome in this meta-analysis.

This review involved a systematic assessment of mainly Chinese-sourced studies reporting immunological serum markers in patients with psoriasis vulgaris of blood-heat syndrome compared to healthy controls; 17 studies were identified for systematic review and meta-analysis. Although most studies used small sample sizes, analysis of the pooled data showed an elevated MD of serum IFN-*γ*, IL-17, IL-23, and TNF-*α* in patients with psoriasis vulgaris of blood-heat syndrome, when compared to the control groups. Pooled IL-4 and IL-10 levels were significantly lower in patients than in controls, but pooled IL-6 levels were not significantly elevated. A simplified model depicting the role of the immunological markers of blood-heat syndrome is presented in Figure 3.

### 4.2. Possible Rationales

According to TCM, patients with psoriasis vulgaris present with one of three syndromes: blood-heat syndrome (53.8%), blood-dryness syndrome (27.4%), or blood-stasis syndrome (18.1%) [9]. At the initiation of the active stage, psoriasis vulgaris usually manifests as blood-heat syndrome; later it may be ameliorated or be converted to blood-dryness/blood-stasis syndrome. The clinical efficacy of TCM, including internal and external applications, in treating psoriasis vulgaris of blood-heat syndrome has been confirmed by a number of previous studies [29–32]. Specifically, the treatment principles of clearing heat and cooling blood are believed to have a positive influence on various pathogenic mechanisms observed in psoriasis because of their anti-inflammatory and antiangiogenic effects, as well as their potential to adjust the Th1/Th2 equilibrium and to change the cytokine balance [15, 26].

Each T-cell subset produces distinct cytokine expression profiles that influence cell fate specification. These different cytokines, released within an inflammatory context, may contribute to psoriasis susceptibility and pathogenesis. Th1 cells develop in the presence of IL-12 and mainly produce IFN-*γ*, IL-2, and lymphotoxin. Th2 cells differentiate in the presence of IL-4 and produce IL-4, IL-5, and IL-13. In the presence of IL-6 and IL-23, Th17 cells are characterized by their capacity to generate cytokines such as IL-6, IL-17, IL-22, and CCL20 [33, 34]. It has been shown that serum levels of IFN-*γ* are much higher in patients with psoriasis than in controls and were correlated with the PASI score (psoriasis activity and severity index), whereas levels of Th2 cytokines (IL-4 and IL-10) were reported to be lower [35, 36]. An important cytokine that is generated by Th17 cells, IL-17F, shows significantly higher mRNA expression in lesional skin, and serum levels of the IL-17F protein were substantially increased in a psoriasis(-like) mouse model as well [37–39]. The cytokines IL-6 and TNF-*α*, which are produced by keratinocytes, play an important role in the activation of innate immunity through activation of dendritic and T-cells [40].

### 4.3. Limitations of This Review

Our analysis has some limitations. First, we were unable to measure the progression of psoriasis vulgaris of blood-heat syndrome using serum markers in pretherapeutic patients, and only three/four eligible observation studies that reported serum IL-23/IL-6 levels were reviewed. We used two different methods to assess publication bias, and, based on the method used, we found some extent of publication bias. The distorting effects of publication and location bias on systematic reviews and meta-analyses have been well documented [41]. Although we are confident that our search strategy enabled us to locate all relevant studies, a certain degree of uncertainty nevertheless remains. Furthermore, the 17 included studies were mainly observational and consisted of small numbers of patients with psoriasis vulgaris of blood-heat syndrome; more high-quality studies, with a low risk of bias and adequate sample sizes, are required to fully clarify the effects.

## 5. Conclusion

In summary, patients with psoriasis vulgaris of blood-heat syndrome show significantly elevated levels of IFN-*γ*, IL-17, IL-23, and TNF-*α* and decreased levels of IL-4 and IL-10. To investigate the clinical relevance of these findings, a review summarizing the evidence on the effect of clearing heat and cooling blood therapy on markers of immunology would be useful.

## Supplementary Material

The methods and findings of the present review have been reported following the MOOSE Checklist. Funnel plots identifying publication bias for all studied outcomes were included in this MOOSE Checklist.

## Figures and Tables

**Table 1:** Characteristics of the included studies and NOS quality assessment.

Author (pub. year)	Study setting	Study period MM/YY–MM/YY	Psoriasis, blood heat syndrome					Healthy control			NOS quality assessment			
			*N*	Mean age, years, mean (SD)	Males (%)	Mean PASI (SD)	Duration, years, mean (SD)	*N*	Mean age, years, mean (SD)	Males (%)	Selection	Comparability	Exposure/outcome	Overall star rating
Cao et al. (2014) [10]	China	07/2011–04/2012	16	46.36 (13.04)	63.33	10.35 (5.84)	NR	16	27.31 (1.40)	56.67	++		+++	5
Chen et al. (2014) [11]	China	09/2011–04/2012	15	46.20 (13.97)	53.33	15.8 (8.60)	3.26 (2.98)	16	42.31 (13.61)	53.33	+	++	+++	6
Chen and Yang (2012) [23]	China	01/2010–12/2010	17	31.00 (1.50)	52.94	24.21 (7.03)	7–10	15	30.00 (0.50)	53.33	+	++	+++	6
Fan et al. (2015) [12]	China	11/2010–10/2011	30	37.50 (7.50)	53.33	11.05 (9.10)	0–30	10	40.29 (9.91)	70.00	+	++	+++	6
He et al. (2015) [13]	China	09/2012–09/2013	40	35.92 (12.91)	62.50	18.619 (3.403)	5.53 (5.25)	20	33.30 (10.96)	60.00	+	++	+++	6
Hu and Yang (2015) [24]	China	NR	30	NR	54.44	NR	NR	30	Match	50.00	+	++	+++	6
Li (2010) [14]	China	03/2009–03/2010	30	41.59 (14.73)	70.59	10.99 (11.62)	NR	18	NR	NR	+		+++	4
Li (2011) [15]	China	04/2009–08/2010	24	NR	NR	NR	NR	20	Match	Match		++	+++	5
Lin et al. (2008) [16]	China	10/2005–03/2006	30	33.13 (11.10)	70.00	10.78 (3.51)	10.47 (9.02)	20	Match	Match	+	++	+++	6
Liu (2011) [17]	China	10/2009–10/2010	20	42.45 (13.55)	65.00	12.80 (4.30)	11.98 (10.23)	20	37.05 (14.16)	40.00		++	+++	5
Liu et al. (2012) [18]	China	03/2010–12/2010	20	34.11	60.00	NR	NR	10	30.50	50.00		++	+++	5
Sun and Zhao (2010) [19]	China	10/2008–02/2010	30	33.00 (13.57)	43.33	21.42 (3.06)	6.70 (5.24)	18	31.72 (12.06)	44.44	+	++	+++	6
Wu et al. (2013) [25]	China	NR	30	NR	NR	NR	NR	30	NR	NR	+		+++	4
Zhang (2003) [20]	China	05/2001–01/2003	20	NR	NR	NR	NR	10	NR	NR			+++	3
Zhang et al. (2010) [21]	China	06/2006–05/2009	47	Match	56.00	NR	16.67–34	31	41.50 (9.20)	67.742		++	+++	5
Zhou (2007) [22]	China	06/2006–03/2007	14	Match	Match	NR	NR	31	41.50	64.50		++	+++	5
Zhou and Wang (2011) [26]	China	10/2009–04/2011	30	32.21 (8.33)	70.00	8.92 (2.07)	16.67–32	10	Match	Match	++	++	+++	7

PASI, Psoriasis Area and Severity Index; NR, not reported; NOS, Newcastle-Ottawa Scale; NOS quality assessment: a star system was used to allow a semiquantitative assessment of study quality. A study could be awarded a maximum of one star for each numbered item within the selection and exposure categories. A maximum of two stars could be awarded for comparability. The NOS ranges from zero to nine stars. We consider those that achieve seven or more stars as high-quality studies, those that achieve four to six stars as medium-quality studies, and those that achieve fewer than four stars as poor-quality studies.

**Table 2:** Summary of immunological markers of blood-heat syndrome.

Source	Markers used ELISA measurement methods, mean (SD)						
	IFN-*γ* (pg/mL)	IL-4 (pg/mL)	IL-17 (pg/mL)	IL-23 (pg/mL)	IL-6 (pg/mL)	TNF-*α* (pg/mL)	IL-10 (pg/mL)
Cao et al. [10]	Control 47.44 (21.46); psoriasis 91.20 (48.37)	Control 47.41 (14.26); psoriasis 32.73 (7.90)	Control 49.15 (31.18); psoriasis 60.30 (48.08)				

Chen et al. [11]	Control 46.00 (0.67); psoriasis 86.48 (46.44)	Control 48.45 (13.63); psoriasis 32.52 (8.13)					

Chen and Yang [23]			Control 20.70 (13.20); psoriasis 81.40 (15.10)	Control 201.00 (78.00); psoriasis 529.00 (202.00)			

Fan et al. [12]			Control 2.85 (0.64); psoriasis 7.32 (2.01)	Control 814.93 (372.51); psoriasis 1491.80 (464.36)	Control 109.32 (7.22); psoriasis 418.05 (108.83)		

He et al. [13]		Control 30.07 (3.36); psoriasis 11.29 (3.70)				Control 7.40 (1.56); psoriasis 30.58 (7.14)	

Hu and Yang [24]	Control 8.72 (2.04); psoriasis 24.10 (8.13)	Control 30.21 (3.40); psoriasis 19.89 (1.19)				Control 74.30 (24.14); psoriasis 130.26 (21.99)	

Li [14]	Control 69.16 (29.99); psoriasis 98.68 (49.65)					Control 86.65 (41.26); psoriasis 131.24 (41.24)	Control 85.73 (27.96); psoriasis 105.60 (35.27)

Li [15]						Control 6.43 (0.33); psoriasis 15.29 (0.49)	Control 18.25 (1.44); psoriasis 6.51 (0.97)

Lin et al. [16]	Control 8.72 (2.06); psoriasis 24.10 (7.84)						Control 21.27 (4.80); psoriasis 8.59 (4.97)

Liu [17]	Control 10.86 (5.41); psoriasis 14.19 (6.84)	Control 67.65 (17.01); psoriasis 63.69 (20.23)	Control 46.89 (9.13); psoriasis 55.43 (13.76)				Control 24.39 (7.57); psoriasis 16.84 (6.74)

Liu et al. [18]			Control 6.24 (1.66); psoriasis 23.37 (2.40)	Control 6.95 (0.83); psoriasis 23.12 (1.89)			

Sun and Zho [19]					Control 8.63 (0.88); psoriasis 11.37 (2.80)	Control 21.65 (1.53); psoriasis 39.44 (6.11)	

Wu et al. [25]			Control 46.69 (11.43); psoriasis 114.60 (19.78)				Control 24.39 (2.27); psoriasis 14.14 (1.62)

Zhang [20]						Control 7.37 (0.85); psoriasis 26.67 (17.78)	

Zhang et al. [21]	Control 8.59 (2.59); psoriasis 11.50 (4.48)				Control 78.20 (43.00); psoriasis 239.06 (159.11)	Control 6.5 (3.1); psoriasis 12.8 (7.4)	

Zhou [22]					Control 71.43 (45.90); psoriasis 244.41 (133.25)	Control 5.9 (3.4); psoriasis 11.3 (7.8)	

Zhou and Wang [26]	Control 33.34 (4.50); psoriasis 96.14 (12.29)	Control 25.91 (3.69); psoriasis 12.88 (2.66)				Control 8.09 (1.06); psoriasis 32.64 (6.23)	Control 21.96 (3.16); psoriasis 12.77 (2.70)

ELISA, enzyme-linked immunosorbent assay; SD, standard deviation; IFN, interferon; IL, interleukin; TNF, tumor necrosis factor.
