# Supplementary material for: Immune Signatures in Patients with Psoriasis Vulgaris of Blood-Heat Syndrome: A Systematic Review and Meta-Analysis
Source: Evid Based Complement Alternat Med. 2016 May 4;2016:9503652. doi: 10.1155/2016/9503652 (PMC4870353; doi:10.1155/2016/9503652)

MOOSE Checklist

Immune signatures in patients with psoriasis vulgaris of blood-heat syndrome: a systematic review and meta-analysis

Xin Li[1,2], Qing-qing Xiao[1,2], Fu-lun Li[1,2], Rong Xu[1,2], Bin Fan[1,2], Min-feng Wu[1,2], Min Zhou[1,2], Su Li[1,2], Jie Chen[1,2], Shi-guang Peng[3*], Bin Li[1,2*]

1. Department of Dermatology, Yueyang Hospital of Integrated Traditional Chinese and Western Medicine, Shanghai University of Traditional Chinese Medicine, Shanghai, China, 200437
2. Institute of Dermatology, Shanghai Academy of Traditional Chinese Medicine, shanghai, China, 201203
3. Department of Dermatology, Beijing Chao-yang Hospital, Capital Medical University, Beijing, China, 100020

* Corresponding author:

Shi-guang Peng: 18501367719@163.com or Bin Li: 18930568129@163.com

Criteria		Brief description of how the criteria were handled in the meta-analysis
**Reporting of background should include**		
√	Problem definition	Page 3: Psoriasis is a common, chronic, relapsing immune-mediated inflammatory disease. Psoriasis prevalence is on the rise, while studies investigating systemic immunity in patients with psoriasis vulgaris of blood-heat syndrome have used different serum markers and reported discrepant results.
√	Hypothesis statement	Page 4: The immunological serum markers IFN-γ, IL-17, IL-23, TNF-α are elevated and IL-4, IL-10 are decreased in patients with psoriasis vulgaris of blood-heat syndrome compared with healthy controls.
√	Description of study outcomes	Page 4: IFN-γ, IL-4, IL-17, IL-23, IL-6, TNF-α, and IL-10
√	Type of exposure or intervention used	Page 4: psoriasis vulgaris of blood-heat syndrome
√	Type of study designs used	Page 5: There were no limitations on the study design.
√	Study population	Page 5: We placed no restriction.
**Reporting of search strategy should include**		
√	Qualifications of searchers	Page 5: The credentials of the three investigators X.L.,

		Q.Q.X, and F.L.L. are indicated in the author list.
√	Search strategy, including time period included in the synthesis and keywords	Page 5: Time period: from January 1980 to May 2015. Keywords: psoriasis, blood-heat syndrome, IFN-γ, IL-4, IL-17, IL-23, IL-6, TNF-α, and IL-10.
√	Databases and registries searched	Page 5: MEDLINE, Embase, Cochrane Central Register of Controlled Trials, China National Knowledge Infrastructure database (CNKI), Chinese Scientific Journals Full-Text Database (CQVIP), Wanfang Data Knowledge Service Platform, and Chinese Biomedical Literature Service System (SinoMed)
√	Search software used, name and version, including special features	Page 5: We did not employ a search software. EndNote was used to merge retrieved citations and eliminate duplications.
√	Use of hand searching	Pages 5-6: We hand-searched bibliographies of retrieved papers for additional references.
√	List of citations located and those excluded, including justifications	Pages 5-6: Details of the literature search process are outlined in the flow chart. The citation list is available upon request.
√	Method of addressing articles published in languages other than English	Page 5: We limited to the studies published in English or Chinese.
√	Method of handling abstracts and unpublished studies	Page 6: We have contacted authors for abstracts and unpublished studies.

√	Description of any contact with authors	Page 6: We have contacted with J. Liu and J.W. Li for their full texts, as adequate information for the performance of this review was unavailable from abstracts.
**Reporting of methods should include**		
√	Description of relevance or appropriateness of studies assembled for assessing the hypothesis to be tested	Pages 5-6: Detailed inclusion and exclusion criteria were described in the study selection section.
√	Rationale for the selection and coding of data	Page 6: Data extracted from each of the studies were relevant to the first author, study characteristics, characteristics of participants, outcome characteristics.
√	Assessment of confounding	Not applicable.
√	Assessment of study quality, including blinding of quality assessors; stratification or regression on possible predictors of study results	Page 6: The Newcastle-Ottawa Scale was used to assess the study quality, by categorizing it into three dimensions: selection, comparability and exposure for case-control studies; and selection, comparability and outcome for cohort studies.
√	Assessment of heterogeneity	Pages 6-7: Heterogeneity of the studies were explored within two types of study designs using I^2 test that provides the relative amount of variance of the summary effect due to the between-study heterogeneity.

√	Description of statistical methods in sufficient detail to be replicated	Pages 6-7: Description of methods of meta-analyses was detailed in the data synthesis and analysis section.
√	Provision of appropriate tables and graphics	We included 1 flow chart, 1 summary tables and 4 figures.
**Reporting of results should include**		
√	Graph summarizing individual study estimates and overall estimate	Figure 3
√	Table giving descriptive information for each study included	Table 1
√	Results of sensitivity testing	Not applicable.
√	Indication of statistical uncertainty of findings	Pages 8-9: 95% confidence intervals were presented with all summary estimates.
**Reporting of discussion should include**		
√	Quantitative assessment of bias	Assessed using funnel plots (see below)
√	Justification for exclusion	Pages 10-11: Papers were excluded on the basis of exclusion criteria listed. We did not systematically exclude any studies on the basis of language or study population size.

√	Assessment of quality of included studies	The Newcastle-Ottawa Scale was used to assess the study quality. Details are outlined in Table 1.
**Reporting of conclusions should include**		
√	Consideration of alternative explanations for observed results	Page 11: We discussed the limitations of this study. We were unable to measure the progression of psoriasis vulgaris of blood-heat syndrome using serum markers in pretherapeutic patients, and only three/four eligible observation studies that reported serum IL-23/IL-6 levels were reviewed. We noted that more high-quality studies, with a low risk of bias and adequate sample sizes, are required to fully clarify the effects.
√	Generalization of the conclusions	Page 12: Patients with psoriasis vulgaris of blood-heat syndrome show significantly elevated levels of IFN-γ, IL-17, IL-23, and TNF-α and decreased levels of IL-4 and IL-10.
√	Guidelines for future research	Page 11: We recommend future studies on the effect of clearing heat and cooling blood therapy on markers of immunology.
√	Disclosure of funding source	Page 16: Description of the detail in the acknowledgments section.

Supplementary Figures 1 a to g. Funnel plots identifying publication bias for all studied outcomes (Funnel plot of MD for included studies).

a. IFN-γ

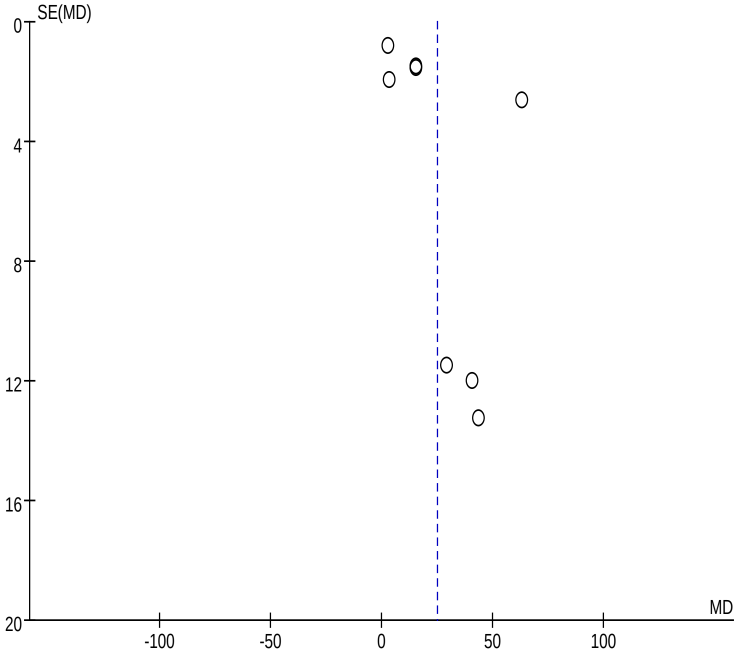

b. IL-4

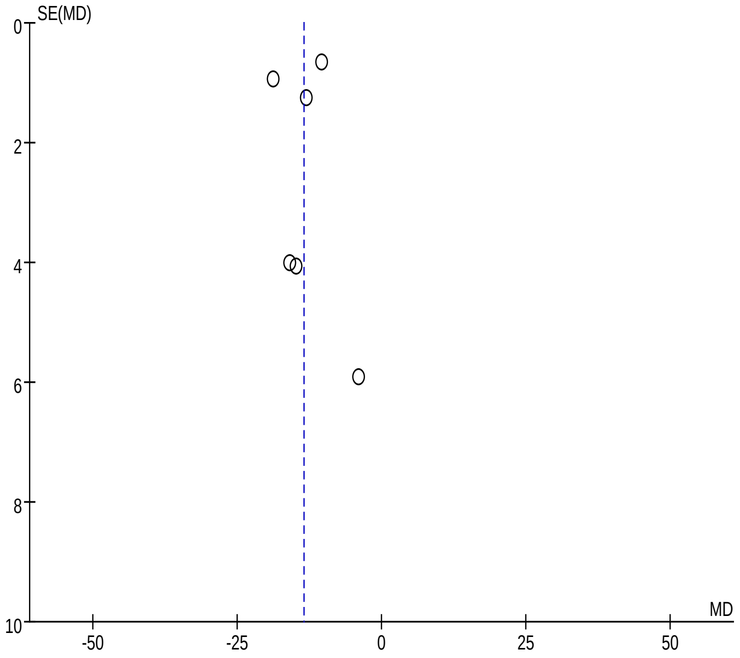

c. IL-17

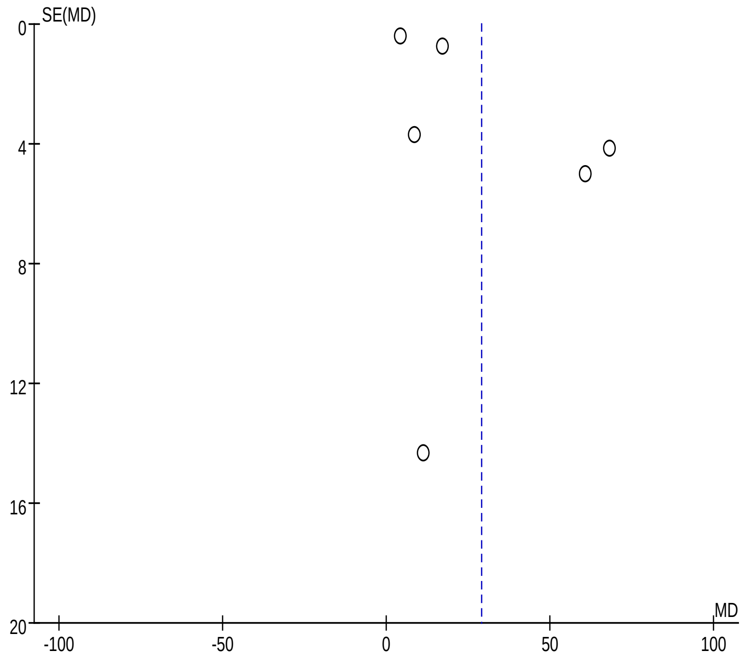
SE(MD)
0
4
8
12
16
20
-100
-50
0
50
100
MD

d. IL-23

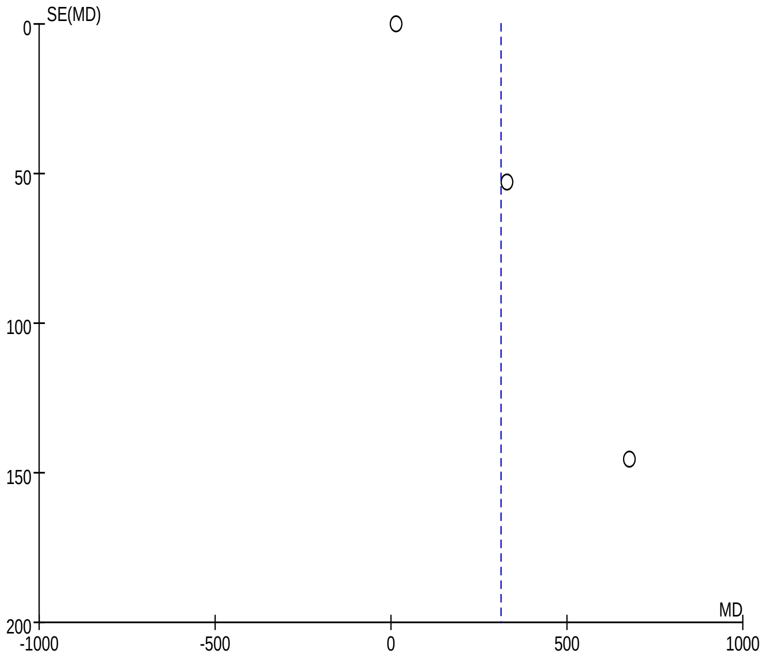
SE(MD)
0
50
100
150
200
-1000
-500
0
500
1000
MD

e. IL-6

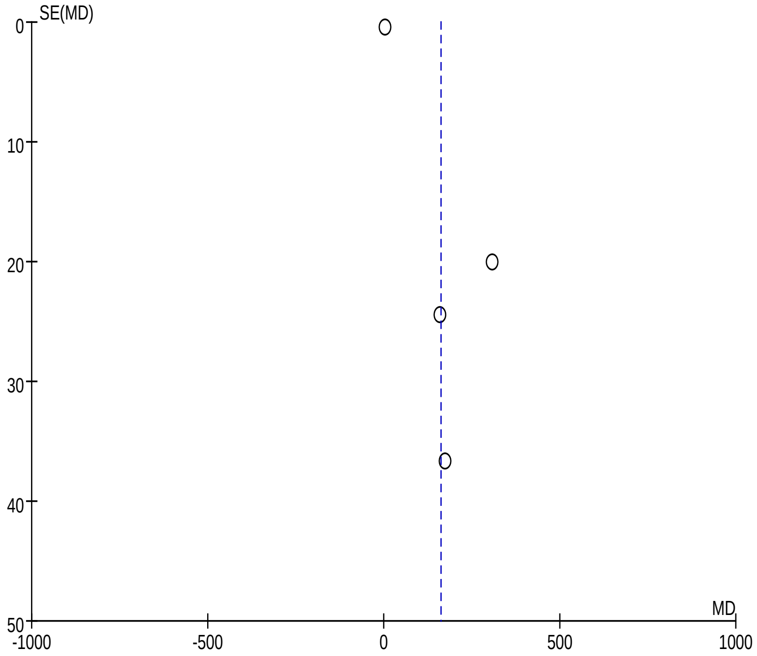

SE(MD)
0
10
20
30
40
50
MD
-1000
-500
0
500
1000

f. TNF-α

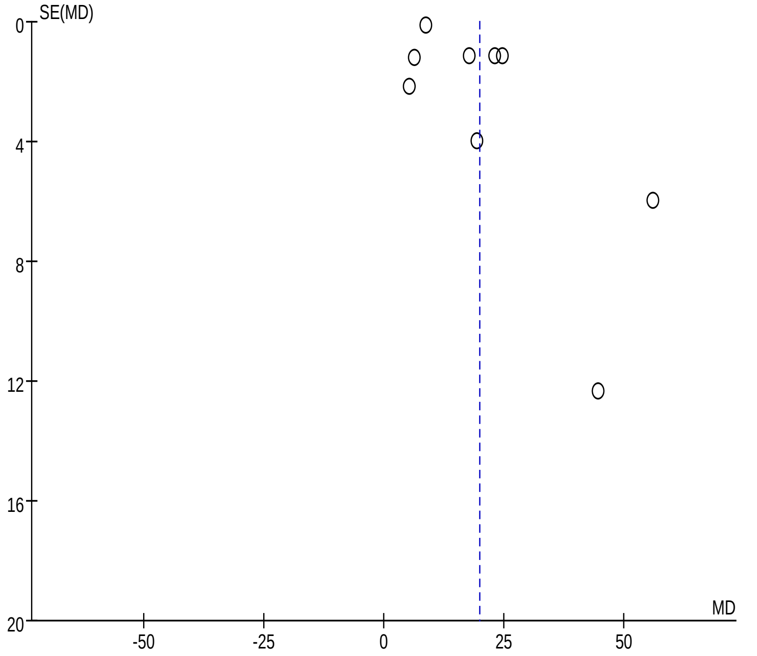

SE(MD)
0
4
8
12
16
20
MD
-50
-25
0
25
50

g. IL-10

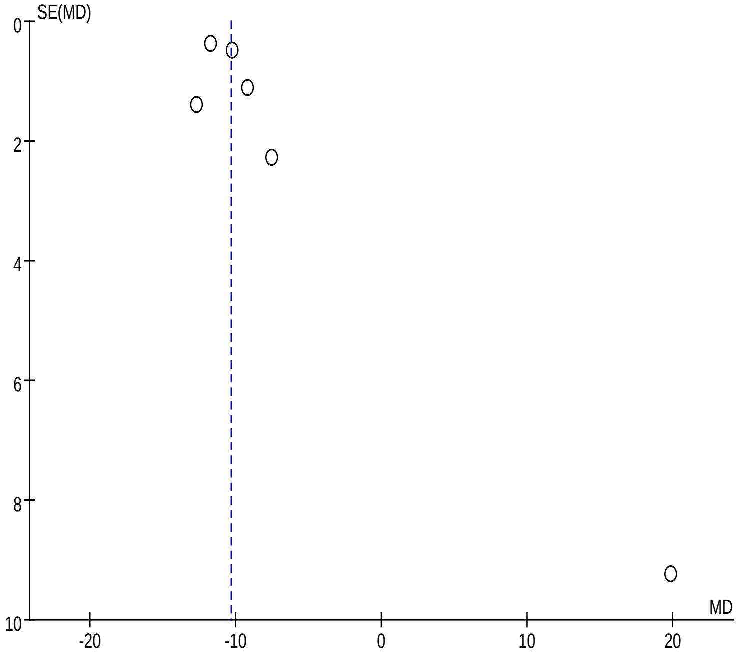

Supplement: Supplementary file 1 — The methods and findings of the present review have been reported following the MOOSE Checklist. Funnel plots identifying publication bias for all studied outcomes were included in this MOOSE Checklist. [file 9503652.f1.pdf]
